# Thermally activated delayed fluorescence processes for Cu(i) complexes in solid-state: a computational study using quantitative prediction†

**DOI:** 10.1039/c8ra04978e

**Published:** 2018-08-09

**Authors:** Lingling Lv, Kui Liu, Kun Yuan, Yuancheng Zhu, Yongcheng Wang

**Affiliations:** College of Chemical Engineering and Technology, Tianshui Normal University TianShui GanSu 741001 China lvling100@163.com; College of Chemistry and Chemical Engineering, Northwest Normal University LanZhou GanSu 730070 China ycwang02@163.com

## Abstract

The photophysical properties of four representative Cu(i) complex crystals have been investigated using the combination of an optimally tuned one- and two-dimensional range-separated hybrid functional 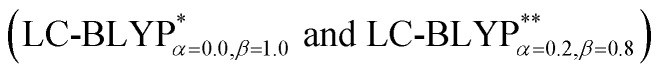 with the polarizable continuum model, and the thermal vibration correlation function (TVCF) approach. The calculated excited singlet–triplet energy gap, radiative rates and lifetimes match the experimentally available data perfectly. At 300 K, the reverse intersystem crossing (RISC) proceeds at a rate of *k*^dir.^_RISC_ ≈ 10^6–8^ s^−1^, which is 4–5 orders of magnitude larger than the mean phosphorescence rate, *k*_P_ ≈ 10^2–3^ s^−1^. At the same time, the ISC rate *k*^dir.^_ISC_ ≈ 10^9^ s^−1^ is again 2 orders of magnitude larger than the fluorescence rate *k*_F_ ≈ 10^7^ s^−1^. In the case of *k*^dir.^_RISC_ ≫ *k*_F_ and *k*^dir.^_RISC_ ≫ *k*_P_, thermally activated delayed fluorescence should occur. Vibronic spin–orbit coupling can remarkably enhance the ISC rates by the vital “promoting” modes, which can provide crucial pathways to decay. This can be helpful for designing novel excellent TADF Cu(i) complex materials.

## Abstract 2

Calculated fluorescence (*k*_F_), phosphorescence (*k*_P_), and ISC rate constants (*k*^vib.^_ISC/RISC_) with the vibronic spin–orbit coupling at 300 K for Cu(dppb)(pz_2_Bph_2_).

## Graphical abstract

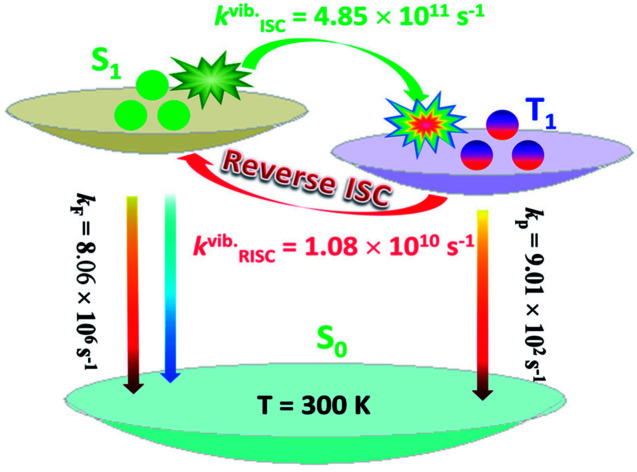

## 1. Introduction

**Fig. 1:**
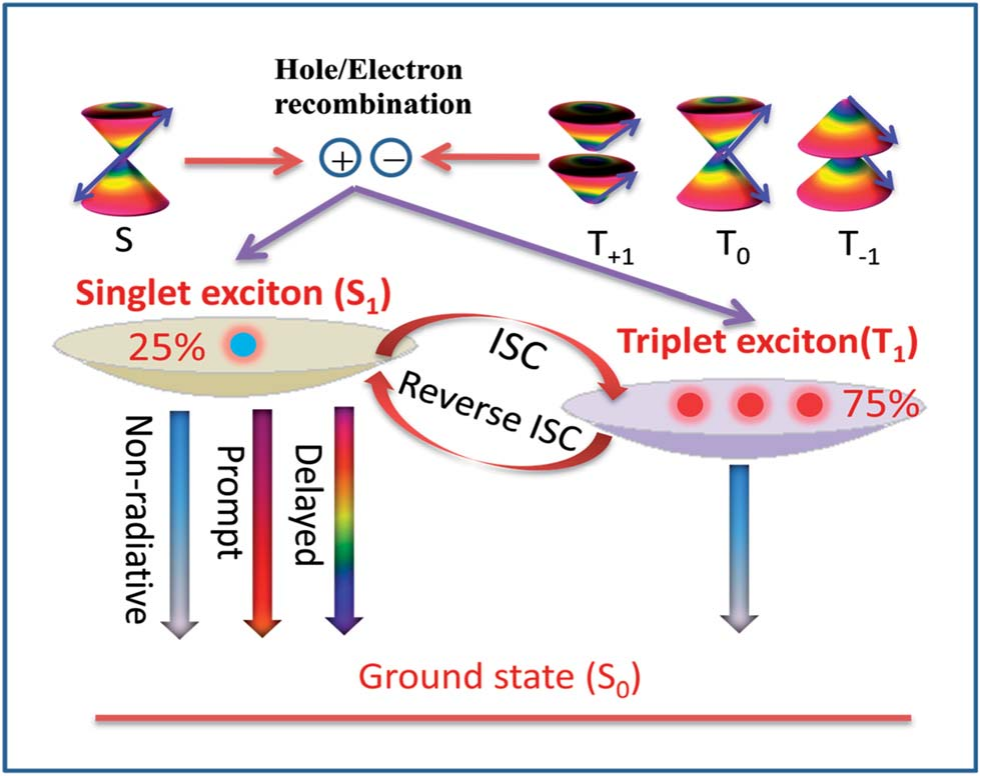
Diagram of the delayed fluorescence process based on spin-statistics in TADF molecules.

Recently, the thermally activated delayed fluorescence (TADF) materials displayed by organo-transition metal complexes and organic molecules have attracted great attention because of the remarkable variability in their emission properties for organic light-emitting diodes (OLED).^1–4^ Under electrical excitation, the singlet–triplet counter pairs of electrons and holes are equally weighted to recombine and yield excitons, resulting in 25% singlet excitons and 75% triplet excitons in the electroluminescence device according to spin statistics.^5–7^ However, the energies of all triplet excitons (75%) are dissipated as heat in the conventional fluorescent materials, which leads to a theoretical upper limit of 25% for the internal quantum efficiency (Fig. 1).^6,7^ To obtain high-efficiency OLED materials, recent studies have found that TADF emitters can rely on efficient thermal upconversion from the triplet state T_1_ into an emissive singlet state S_1_ through reverse intersystem crossing (RISC), which in principle, causes the efficiency of exciton utilization to reach 100%.^8–10^

It is well known that efficient TADF must satisfy the vital condition of a small energy gap, Δ*E*(S_1_–T_1_), between the T_1_ and S_1_ states involved in the RISC process. One effective strategy to decrease the Δ*E*(S_1_–T_1_) involves using covalently linked electron donor and acceptor units and consequently, such molecules have become the focus of molecular designs adopted for TADF.^11^ Using this strategy, one obtains the T_1_ and S_1_ states with strong charge transfer (CT) character from the highest occupied molecular orbital (HOMO) to the lowest unoccupied molecular orbital (LUMO) transitions. These states are characterized by a small spatial overlap between the HOMO and LUMO, which decreases the electron exchange energy and the energy gap, Δ*E*(S_1_–T_1_).^11,12^ However, in this simple model, one negative effect is inevitably generated: the small spatial 〈HOMO|LUMO〉 overlap for S_1_ with CT causes the slow radiative decay rate of the S_1_ → S_0_ transition and low luminescence efficiency. Therefore, it is an essential challenge to obtain a reasonable balance between a small Δ*E*(S_1_–T_1_) and the oscillator strength of the S_1_ → S_0_ transition in the TADF molecular design. An accurate evaluation of Δ*E*(S_1_–T_1_) with a reasonable oscillator strength is necessary to guide the TADF molecular design and expand the range of TADF materials.

Moreover, it is well established that the optical properties of TADF materials can have a considerable impact as a function of their environment. In other words, TADF materials are usually mixed into appropriate host matrixes to remit the concentration quenching and exciton annihilation processes, which restrain the efficiency enhancement of OLEDs. The study reported by Yersin *et al.*^13^ has shown that the same TADF complex can have a much larger photoluminescence quantum yield in the solid state than that in solution. Therefore, it is also a challenge to be able to accurately describe the solid phase excited-state properties of TADF emitters. However, the theoretical studies of TADF materials, taking into account a solid-state environment, have great limitations so far.

From a theoretical standpoint, a reliable and computationally efficient method for the prediction of the excited state properties for the solid state would thus be highly beneficial since it would allow one to possibly help in determining the origin of the variation in experimental results. The time-dependent density functional theory (TD-DFT) is a useful and reliable tool to compute the excited states of relatively larger systems and is considerably more computationally efficient.^14^ Unfortunately, the conventional (semi)local exchange–correlation (XC) functionals may fail completely in predicting the electronic structure in donor–acceptor CT systems.^15^ In addition, for molecular crystals, the surrounding environment is substantially different from that of single molecules, ascribed to polarization effects, which is essentially a phenomenon related to nonlocal correlation. These systematic errors are mainly attributed to the inappropriate XC introduction and the potential and density can be incorrect at asymptotically large distances.^16–18^ Recently, the range-separated exchange (RS) density functional comprising a suitable fixed amount of exact-exchange (eX) has overcome the incorrect asymptotic behavior in the long-range limit, and provides an improved description of the excited-state properties.^16–21^ Abramson *et al.*^22^ reported the dielectric constant (*ε*) in a “screened” RS functional by replacing the 1/*R* asymptotic behavior with the more general asymptotic 1/(*ε* × *R*), which is required when the calculations are performed on the periodic crystals. Very recently, a more computationally efficient method was proposed by Sun *et al.*^23^ for the quantitative characterization of the excited-state properties in molecular crystals. This calculation method is to combine the polarizable continuum model (PCM) and optimally tuned RS functionals, whose advantage comes from the optimal tuning of the range-separation parameter *ω* for a long-range corrected (LC) functional in the PCM environment. For the simulation of a solid-state environment, the molecules in the crystal phase were “dissolved” in a solvent with the same kind of molecules as the crystal.

**Fig. 2:**
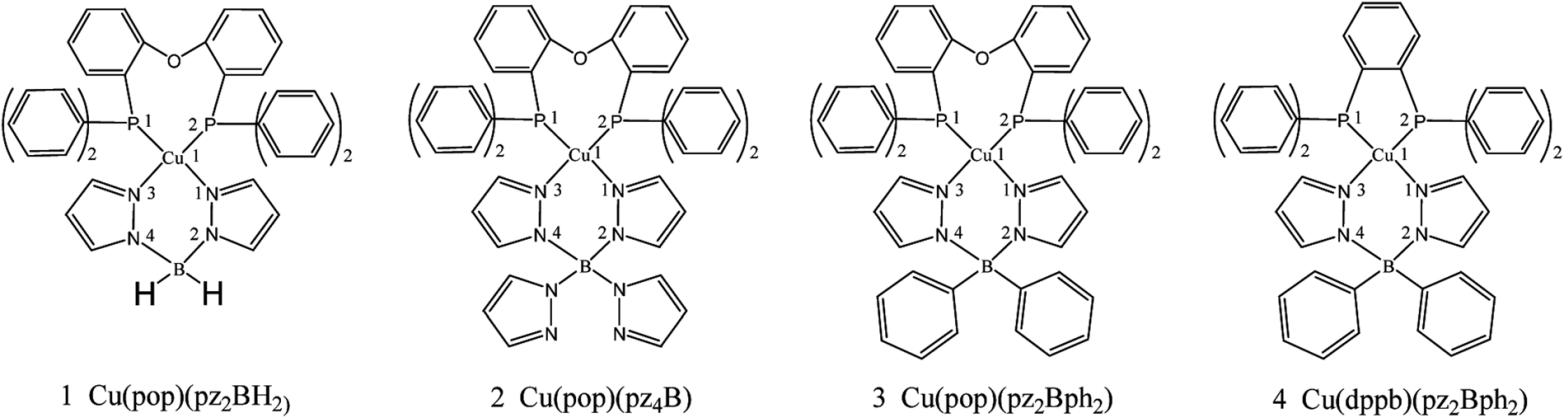
Chemical structure of mononuclear Cu(i) complexes.

Potential candidates for Cu(i) complexes in the fields of OLEDs have attracted a lot of attention from the academic and commercial communities. A much deeper investigation of the photophysical and chemical properties of the Cu(i) compounds will lead to the development of new materials and material design strategies. In this study, we chose four representative TADF emitters that are Cu(i) complexes, namely, Cu(pop)(NN) (pop = bis(2-(diphenylphosphanyl)-phenyl)ether and NN = bis(pyrazol-1-yl)borohydrate(pz_2_BH_2_), tetrakis(pyrazol-1-yl) borate (pz_4_B), bis(pyrazol-1-yl)-diphenyl-borate (pz_2_Bph_2_) and dppb = 1,2-bis(diphenylphosphino)-benzene; Fig. 2), as our research objects because their extensive experimental data are available.^13^ Herein, the *k*_r_ radiative decay rates are determined *via* the Einstein relationship and the *k*_ISC_ decay rates are quantitatively calculated using the thermal vibration correlation function (TVCF) rate theory in combination with the PCM-tuned LC-BLYP method in the solid-state.^24–29^ In addition, vibronic spin–orbit coupling (SOC) has been taken into account from the promoted vibration modes.

## 2. Computation details

### 2.1 Optimization of geometries

The optimal tuning method was performed according to the RS functionals. In the expression of RS hybrid functionals, the two-electron repulsion operator 1/*R*_12_ was divided into a short-range domain and a long-range domain by means of the Ewald-style partition based on the error function (erf) as follows:^30^1
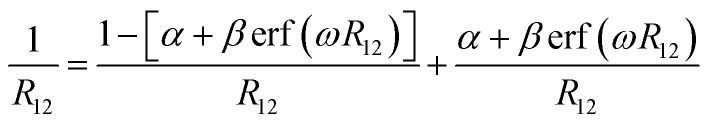
where *R*_12_ is the distance between the electrons at the coordinate vectors *R*_1_ and *R*_2_; the range-separation parameter *ω* (units of bohr^−1^) determines the ratio between the ranges depending on the value of *R*_12_. The first term on the right-hand side in eqn (1) is a short-range interaction described by DFT exchange potential, and the second term is the long-range interaction described by the HF exchange integral. For an asymptotically correct LC functional, *α* + *β* = 1, which is enforced for the optimal tuning in this study. The optimal tuning procedure is based on the exact Kohn–Sham (KS) theory, in which the negative HOMO energy for an N-electron system equals the vertical ionization potential;^23^ this expression is as follows:2
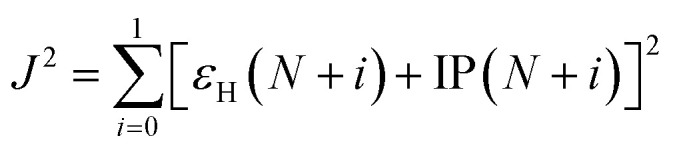


In addition, there have been recent studies^31,32^ showing that some amount of short-range HF exchange (*i.e.* setting *α* to a nonzero value) can lead to improved electronic properties and excitation energies. We used two different parameterizations: a long-range corrected LC-BLYP functional without any short-range exchange (*i.e.*, *α* = 0.0, *β* = 1.0) as well as an LC-BLYP functional containing 20% exchange over the entire range (*i.e.*, *α* = 0.2, *β* = 0.8) in conjunction with tuning *ω via* the non-empirical procedure,^33^ which are denoted as 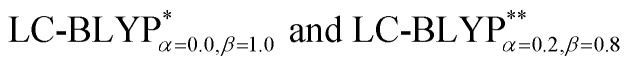.

**Fig. 3:**
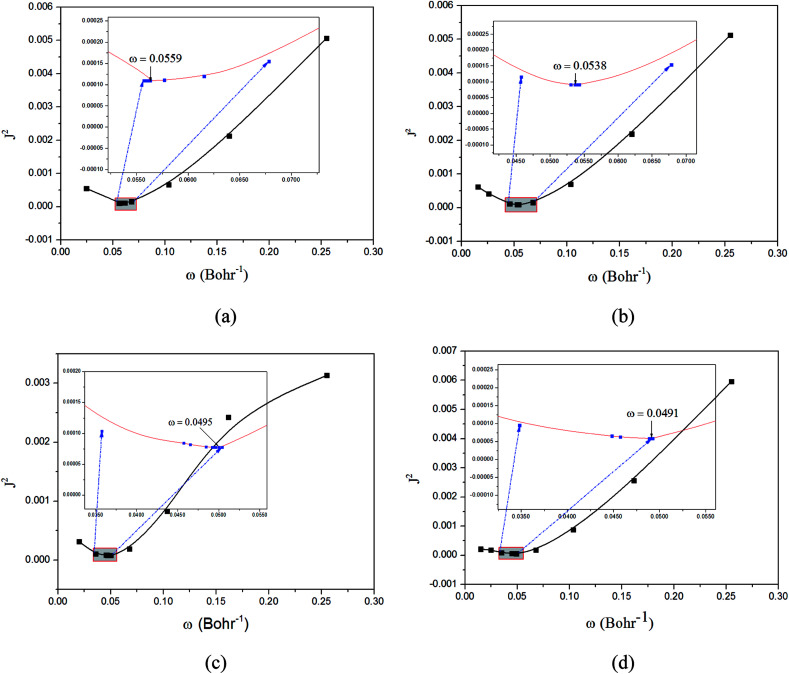
Functions defined in eqn (2) used for determining of the optimally-tuned range-separation parameter *ω* (bohr^−1^) at the 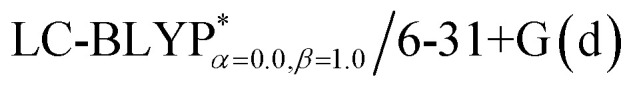 level in the solid state. (a) Cu(pop)(pz_2_BH_2_); (b) Cu(pop)(pz_4_B); (c) Cu(pop)(pz_2_Bph_2_); (d) Cu(dppb)(pz_2_Bph_2_).

The optimization of the parameter *ω* was performed by tuning the optDFTw procedure,^34^ as described in Fig. 3, and all the ground geometries were optimized using the 
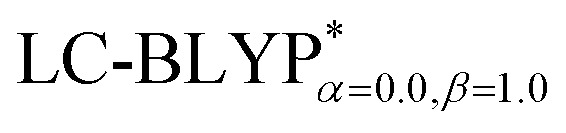
 functional and 6-31+G(d) basis set as shown in Fig. 5. The geometries of the excited states were optimized using the corresponding time-dependent 
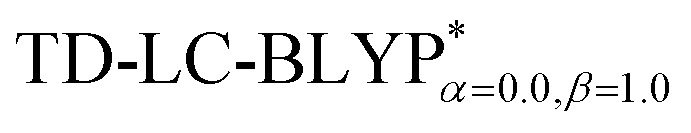
 functionals. As for the simulation of the environmental solid polarization effects, the default PCM model using the integral equation formalism variant was employed, where the molecule in the crystal phase was “dissolved” in the solvent with the same kind of crystal molecules. These were performed by adding the “SCRF (PCM, solvent = generic, read)” keyword in the Gaussian 09 package.^35^ The settings for the PCM parameters have been explicitly described in ref. 23*b*.

**Fig. 4:**
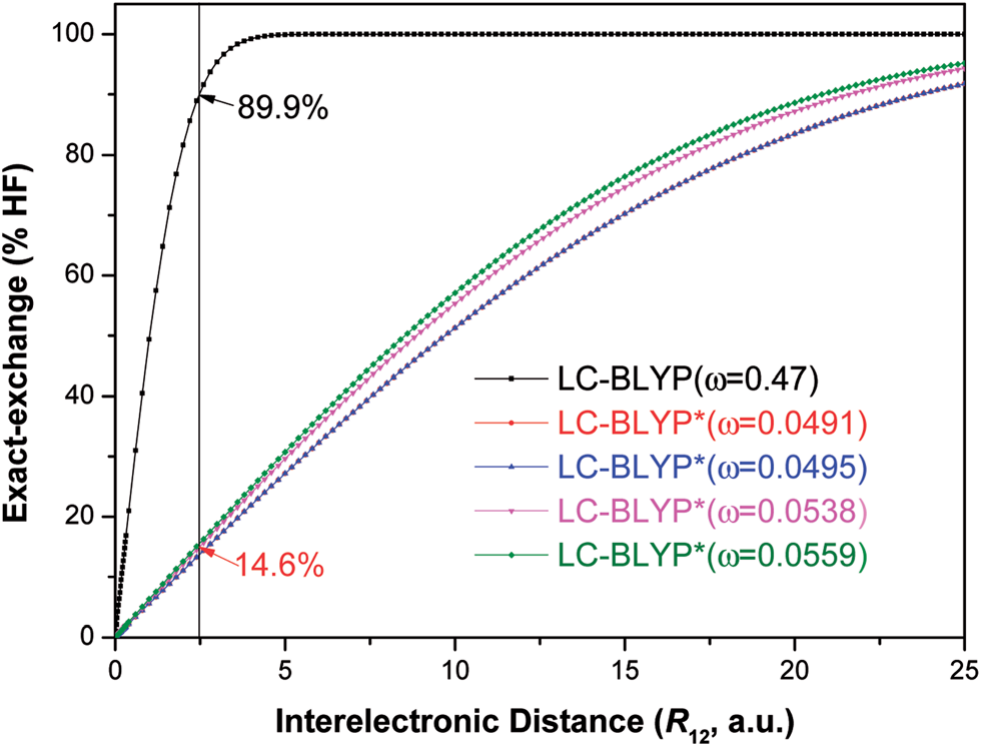
Diagram of the percentage of exact-exchange (%HF) *versus* the interelectronic distance (*R*_12_) for the 
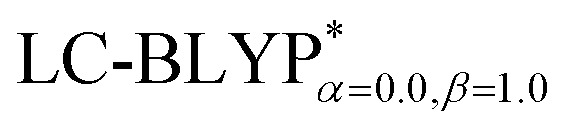
 functional in the four Cu(i) complexes.

**Fig. 5:**
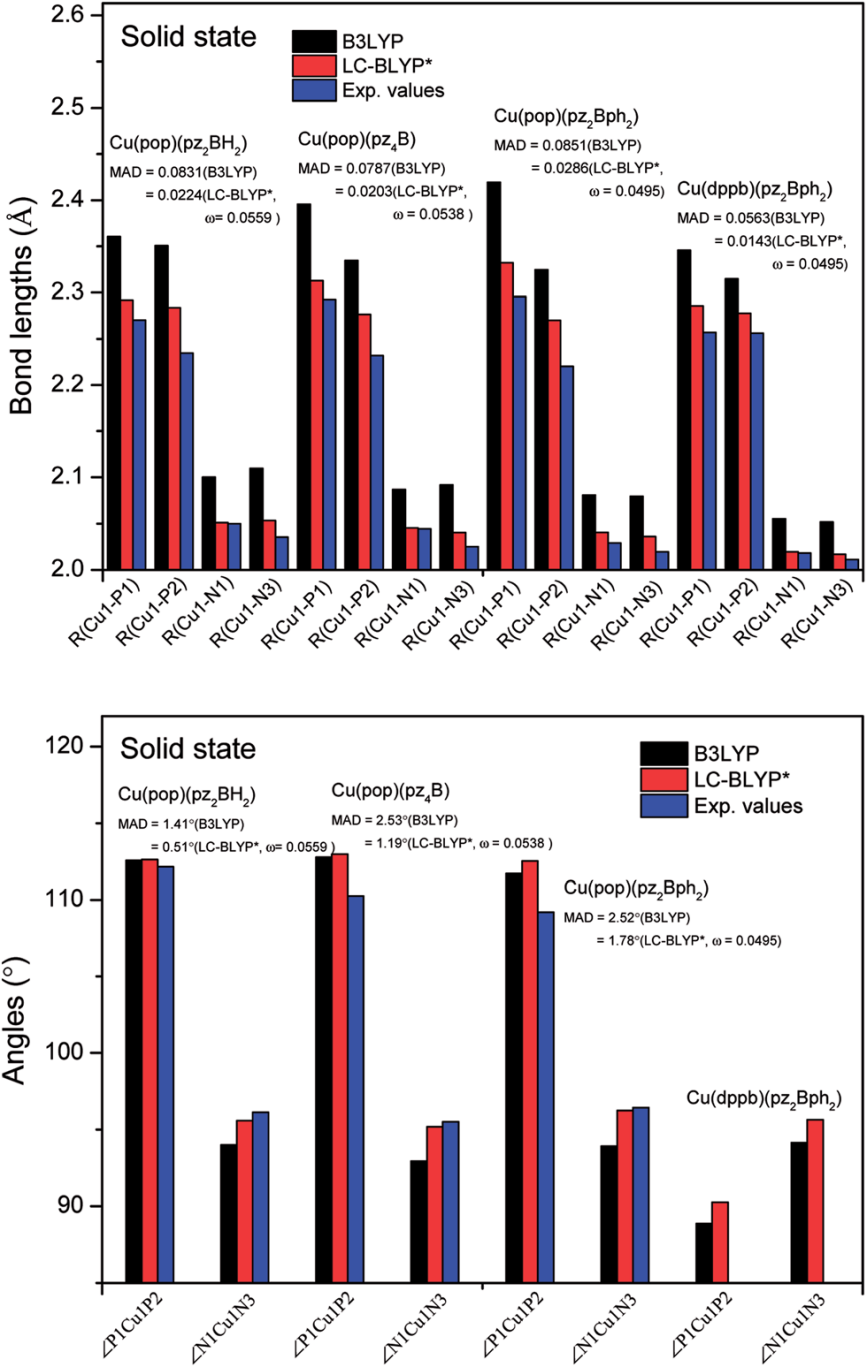
Calculated mean absolute deviations (MAD) of the vital bond lengths and angles at the equilibrium geometries of the ground state S_0_ for the Cu(i) complex molecules in the solid state compared to the experimental values. The MAD values were calculated with respect to the corresponding experimental values, *e.g.*
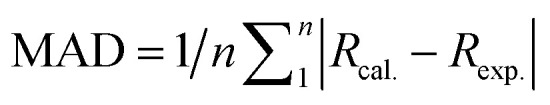
, and 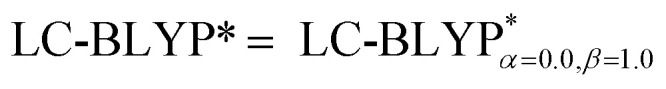.

### 2.2 Calculations of the electronic-structure and energy gap Δ*E*(S_1_–T_1_)

High level of accuracy of calculations is crucial for the quantitative theoretical prediction of TADF molecules. As described in previous studies, the vertical excitation energies of the S_0_ → S_1_, *E*_V_(S_1_) and S_0_ → T_1_, *E*_V_(T_1_) and the energy gap Δ*E*(S_1_–T_1_) = *E*_V_(S_1_) − *E*_V_(T_1_) were calculated by applying the PCM-tuned 
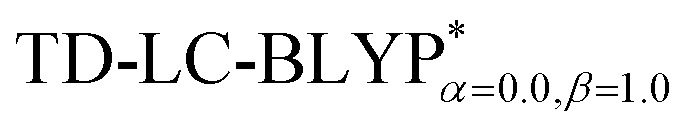
 and PCM-tuned 
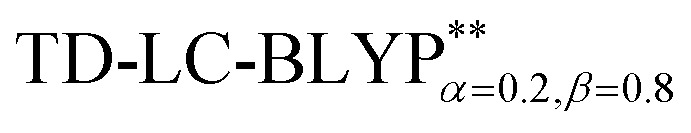
 functionals, combined with the 6-31+G(d) basis set within the Tamm–Dancoff approximation (TDA).^36^ For comparison, two exchange–correlation functionals, namely, B3LYP (*α* = 0.2) and M062X (*α* = 0.54), and two RS hybrid functions, CAM-B3LYP (*ω* = 0.33, *α* = 0.19, *α* + *β* = 0.65) and *ω*B97XD (*ω* = 0.20, *α* = 0.22, *α* + *β* = 1.0), were used.^23*b*,25^ To give a quantitative characterization of the excited states, we also calculated the distance between the centroid of the hole and electron, Δ*R*_H–L_, hole–electron overlap integral *S*_H–L_, and CT excitation energy *E*_CT_ corresponding to the S_0_ → S_1_ transition. These calculations were conducted at the theoretical level of PCM(ε)-tuned 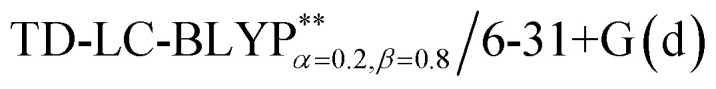 using the Multiwfn program.^37^

### 2.3 Calculations of fluorescence and phosphorescence rates

The phosphorescence transitions (T_1_ → S_0_) are strictly forbidden in the regime of non-relativistic treatment. If the SOC is considered, the pure singlet or triplet becomes a mixed state. In this case, the forbidden emission can borrow dipole activity from spin-allowed transitions (S_0_ ↔ S_*n*_ and T_1,ζ_ ↔ T_*m*,ζ_; see eqn (3)) through the perturbation SOC interactions, resulting in the non-zero intensity of transitions.^38^ These calculations were performed using the parallel version of the combined PCM-tuned 
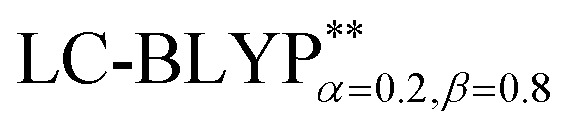
 functional and restricted open-shell configuration interaction with the single excitation (DFT/ROCIS) method by the ORCA 4.0 package.^39^ DFT/ROCIS calculations with the parameters^40^*c*_1_ = 0.21, *c*_2_ = 0.49, and *c*_3_ = 0.29 were performed using the converged restricted Kohn–Sham functions at the PCM-tuned 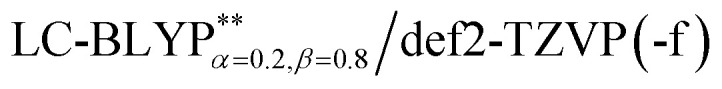 level, together with the auxiliary basis set def2/*J* in order to accelerate the calculations in the framework of the RI approximation. The electric dipole transition, 
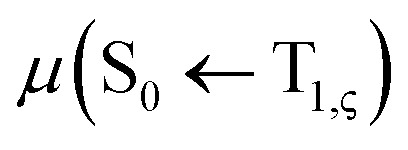
, between T_1_ and S_0_ becomes allowed and 
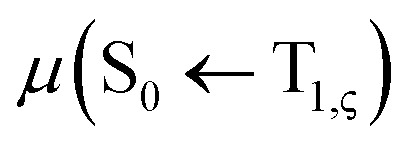
can be expressed as follows:^41,42^3
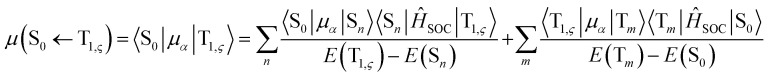
where the operators *μ*_α_ and 
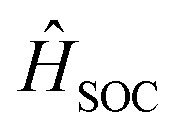
 represent the electric dipole and SOC Hamiltonian, respectively. At ambient temperature, the emission rates can be calculated by eqn (4):^41,42^4
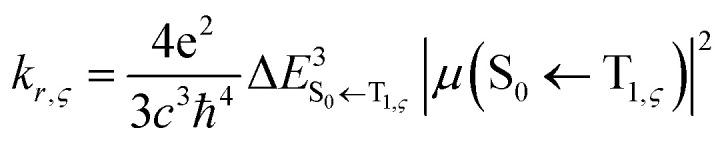
where 
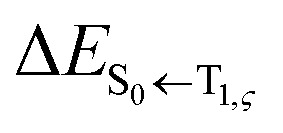
 indicates a vertical emission energy.

### 2.4 Calculations of ISC rates

ISC rates of the T_1_ ↔ S_1_ mainly depend on the energy gap (Δ*E*(S_1_–T_1_)) as well as on the strength of the SOC between T_1_ and S_1_ states. However, in TADF Cu(i) complexes, their SOC interaction is strictly forbidden due to the same CT electronic structures. Therefore, the vibronic SOC contribution to the ISC and RISC rates should be considered,^43^ and these calculations were conducted based on the TVCF theory using the MOMAP suite of programs.^24–29^ The potential energy surface of S_1_ was considered by using Q_S1_ = *S*Q_T1_ + *D*, relative to that of T_1_. The vector *D* is the displacement between S_1_ and T_1_ geometries and *S* is the Duschinsky rotation matrix representing the mixing of normal modes in the S_1_ and T_1_ states; Q represents the nuclear normal mode coordinates. In the Franck–Condon approximation, the ISC rate constant at temperature *T* is then given by eqn (5).^26,44^5
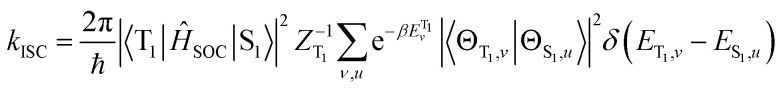
here, 
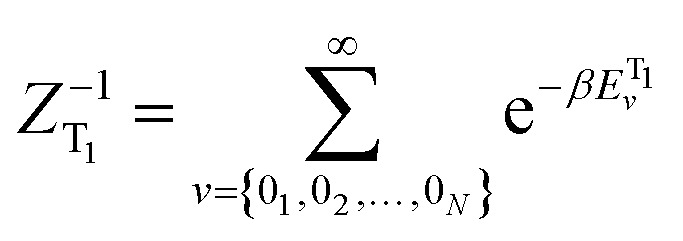
 is the canonical partition function; *v* and *u* are the vibrational quantum numbers of T_1_ and S_1_ states. Further details of the formula can be found in ref. 26.

## 3. Results and discussion

### 3.1 Parameter *ω* optimization and the geometric structures

The optimally tuned derived *ω* values are given in Fig. 3 for the four Cu(i) molecular systems in the solid states; *i.e.*, using the PCM model with the respective screening effects of the dielectric constant of the molecular crystal during the tuning process. Under the environment of the larger dielectric constants, the optimal *ω* values were greatly reduced to the range of 0.0491–0.0559 bohr^−1^ for the solid phase system, which is compared with the default *ω* = 0.47 bohr^−1^ for LC-BLYP. As discussed in detail elsewhere, since *ω* reflects the global delocalization degree, there is an inverse relationship between the tuned *ω* value and the spatial extension of the delocalization. The smaller *ω* values demonstrate that the electron density in the simulated crystal environment is more delocalized than that for the isolated molecule in the gas phase.

In addition, from the perspective of the equation of RS functionals as shown in eqn (1), a smaller *ω* value corresponds to a larger interelectronic distance, *R*_12_, where the description of exchange switches from the short-range DFT-type to the long-range exact-exchange (HF-type) or, in other words, an effective electron delocalization length; the previously optimal *ω* values were found to decrease with increasing system size and conjugation length. However, in some instances, this correlation was not monotonous and varied strongly for systems with different electronic structures. Here, the Cu(i) complexes have different sizes, being smallest for Cu(pop)(pz_2_BH_2_) (*ω* = 0.0559 bohr^−1^) and largest for Cu(pop)(pz_2_Bph_2_) (*ω* = 0.0495 bohr^−1^) and Cu(dppb)(pz_2_Bph_2_) (*ω* = 0.0491 bohr^−1^), which has a strong dependence of *ω* on the size of ligands around the central Cu(i) atom.

To illustrate the above behavior, the dependence of the percentage (%HF) of eX in LC-BLYP and 
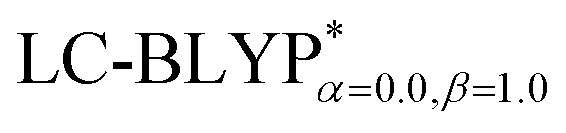
*versus* the *R*_12_ for four Cu(i) complexes is displayed in Fig. 4. We take the tuned *ω* value of the 
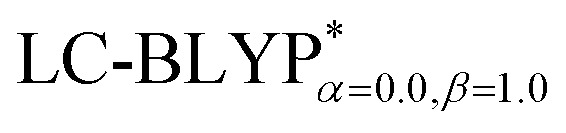
 functional for the Cu(dppb)(pz_2_Bph_2_) system as an example. At *R*_12_ = 2.5 a.u. for the solid state, the HF exchange of the optimally tuned functional is 14.6% (*ω* = 0.0491 bohr^−1^), whereas the default LC-BLYP (*ω* = 0.47 bohr^−1^) gives more than 89.9% HF exchange. This indicates that the precise description of the solid phase requires the functionals to include less “localized” HF exchange and more “delocalized” DFT-BLYP exchange for the Cu(i) complexes. The eX percentage functional of the other three complexes is similar to that of Cu(dppb)(pz_2_Bph_2_).

The ground state (S_0_) geometries of the solid state molecules were optimized using the optimally tuned functional 
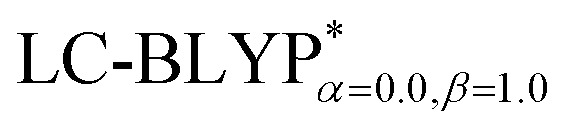
 and conventional functional B3LYP. These structural parameters are illustrated in Fig. 5 and Table S1 in the ESI.† It is easily noted that the optimal tuning of functionals is very important for the geometric optimization of S_0_. For the B3LYP, deviations in the optimized results are much larger. For example, the mean absolute deviations (MADs) in variation between the lower and the upper deviations are 0.0563 Å and 0.0851 Å, respectively, for the bond lengths in the four Cu(i) complexes and between 1.41° and 2.53° for the bond angles, as compared with the crystal data. For the PCM-tuned (*ω* = 0.0559 bohr^−1^) 
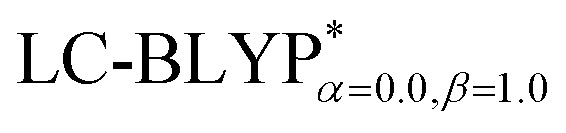
 functional, the MADs were merely 0.0224 Å for the bond lengths and 0.51° for the bond angles in the Cu(pop)(pz_2_BH_2_) complexes; these estimated geometric parameters are in good agreement with experimental crystal data. In this study, we ultimately chose the PCM-tuned (TD-) 
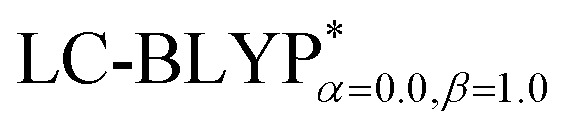
 level, unless otherwise stated, to optimize the geometry structures (including the S_0_, S_1_ and T_1_ states) of the Cu(i) molecular crystals.

### 3.2 Energy gap Δ*E*(S_1_–T_1_) and transition properties

A concise summary of all four Cu(i) molecule excited energies obtained by TD-DFT (B3LYP, M062X, ωB97XD, CAM-B3LYP, 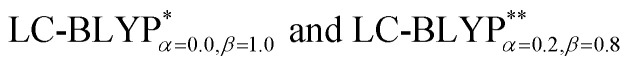) is given in Table 1. Overall, the two RS functionals provide significantly better predictions for the excited state energies and properties than the conventional hybrid functionals. Compared to the experimental Δ*E*(S_1_–T_1_) values, the energy gaps of Cu(i) molecules are most accurately calculated by 
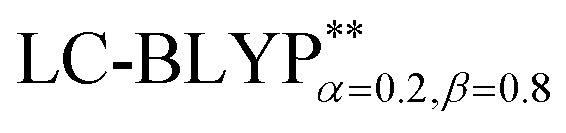
, including some short-range exchange, and least accurately predicted by B3LYP (this can be attributed to the large electron self-interaction error of the B3LYP functional, which spuriously favors the CT character of the excitations). We also found that 
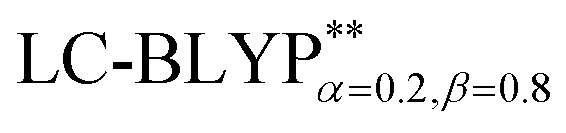
 significantly improves the accuracy of these excitations in comparison to the 
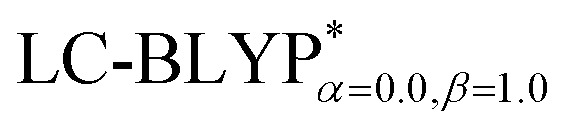
 results. The calculated values of Δ*E*(S_1_–T_1_), 1129, 1048, 887, and 565 cm^−1^ for the four Cu(i) molecules, namely, Cu(pop)(pz_2_BH_2_), Cu(pop)(pz_4_B), Cu(pop)(pz_2_Bph_2_), and Cu(dppb)(pz_2_Bph_2_), are in very good agreement with the measured values of 1300, 1000, 800, and 370 cm^−1^, respectively in the powder.^14^ In the following investigations, 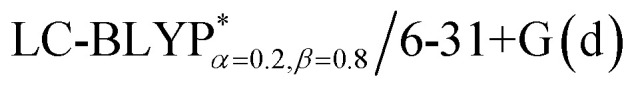 was selected to predict the excited state properties, corresponding to optimal *ω* values listed in Table 2.

**Table 1:** Calculated vertical excited energies (eV), *E*_v_(S_1_) and *E*_v_(T_1_), and the corresponding energy difference Δ*E*(S_1_–T_1_) at the S_0_ minimum using the different functional methods, and compared to the experimental values^a^

Methods	Cu(pop)(pz_2_BH_2_)			Cu(pop)(pz_4_B)			Cu(pop)(pz_2_Bph_2_)			Cu(dppb)(pz_2_Bph_2_)		
	*E*_v_(S_1_)	*E*_v_(T_1_)	Δ*E*(S_1_–T_1_)	*E*_v_(S_1_)	*E*_v_(T_1_)	Δ*E*(S_1_–T_1_)	*E*_v_(S_1_)	*E*_v_(T_1_)	Δ*E*(S_1_–T_1_)	*E*_v_(S_1_)	*E*_v_(T_1_)	Δ*E*(S_1_–T_1_)
B3LYP	3.34	3.20	0.14(1129)	3.34	3.22	0.12(968)	3.27	3.16	0.11(887)	3.03	2.96	0.07(565)
M062X	4.42	4.10	0.32(2581)	4.43	4.03	0.40(3226)	4.39	4.09	0.30(2420)	3.83	3.54	0.29(2339)
ωB97XD	4.29	3.89	0.40(3226)	4.30	3.89	0.41(3307)	4.26	3.88	0.38(3065)	3.83	3.51	0.32(2580)
CAM-B3LYP	4.28	3.81	0.47(3791)	4.29	3.80	0.49(3952)	4.25	3.80	0.45(3629)	3.81	3.50	0.31(2500)
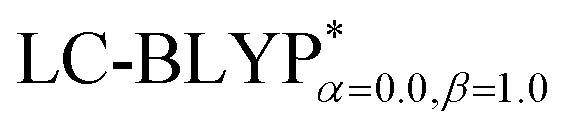	2.51	2.45	0.06(484)	2.52	2.47	0.05(403)	2.42	2.35	0.07(565)	2.28	2.23	0.05(403)
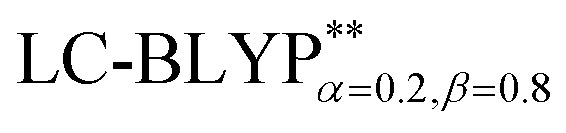	**3.34**	**3.20**	**0.14(1129)**	**3.35**	**3.22**	**0.13(1048)**	**3.27**	**3.16**	**0.11(887)**	**3.03**	**2.96**	**0.07(565)**

a The experimental values of Δ*E*(S_1_–T_1_): 1300, 1000, 800, and 370 cm^−1^ for Cu(pop)(pz_2_BH_2_), Cu(pop)(pz_4_B), Cu(pop)(pz_2_Bph_2_), and Cu(dppb)(pz_2_Bph_2_), respectively; vertical excited energies between about 3.35 and 3.99 eV.

**Table 2:** Optimal range-separated parameters *ω* (bohr^−1^), dielectric constant *ε*, calculated ionization potential (IP), electron affinity (EA), energetic gap (*E*_g_), CT excitation energy (*E*_CT_) of the molecules in the solid phase; energy is in eV

Species	PCM(ε)-tuned 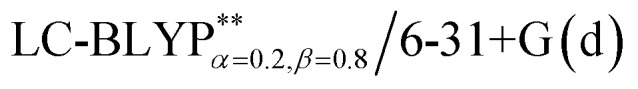					
	*ε*	*ω*	IP(-*ε*_H_)	EA(-*ε*_L_)	*E*_g_	*E*_CT_ a
Cu(pop)(pz_2_BH_2_)	3.19	0.0169	5.01	0.59	4.42	0.38
Cu(pop)(pz_4_B)	3.23	0.0172	5.10	0.68	4.42	0.30
Cu(pop)(pz_2_Bph_2_)	3.49	0.0144	4.93	0.65	4.28	0.14
Cu(dppb)(pz_2_Bph_2_)	3.66	0.0131	4.84	0.78	4.06	−0.63

a The CT excitation energy: *E*_CT_ = IP − EA − 1/*R*; *ε*_H_ = HOMO energy, *ε*_L_ = LUMO energy.

**Fig. 6:**
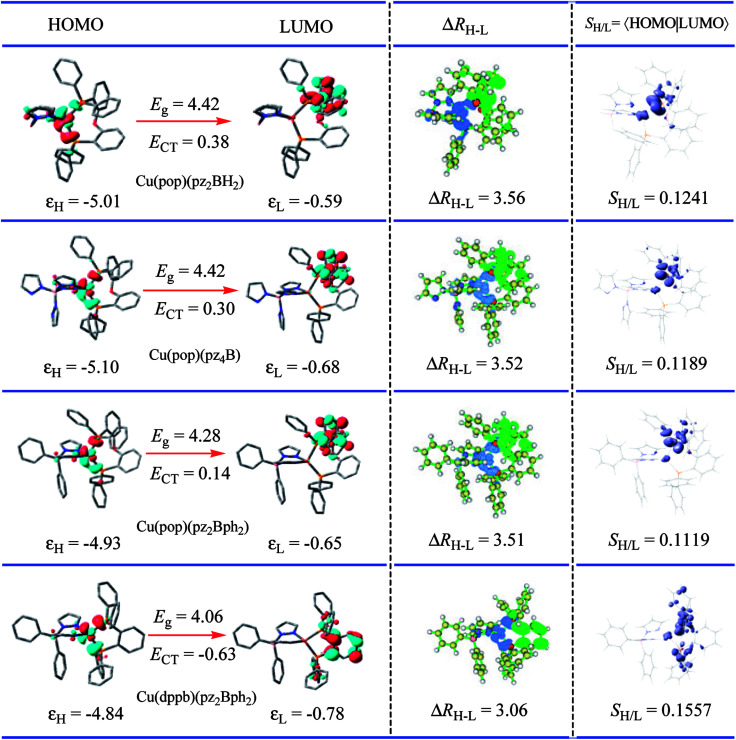
Computed hole (HOMO)-electron (LUMO) indexes of the mononuclear Cu(i) complexes for selected S_1_ states at the PCM(ε)-tuned 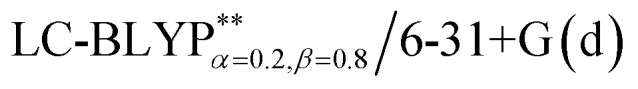 theoretical level. The distance between the centroid of the hole and electron, Δ*R*_H–L_ is in Å, the hole–electron overlap integral *S*_H–L_ is in a.u.; *ε*_H_, HOMO energy; *ε*_L_, LUMO energy; *E*_g_, energetic gap; *E*_CT_, CT excitation energy; all energy units are in eV.

For the studied complexes, their first S_1_ and T_1_ excited states mainly come from the electronic transitions from HOMO to LUMO, and they possess obvious CT character and have the same configurations, ^1^CT and ^3^CT (see Fig. 6). Based on the quantum theory, the S_1_ and T_1_ states are separated by twice the electron exchange energy *J*, as illustrated in eqn (6) and (7) with *ϕ*_L_ and *ϕ*_H_ corresponding to the electron (LUMO) and hole (HOMO) orbitals involved in the transitions, respectively. The *J* value is determined by the spatial separation and overlap integral of *ϕ*_L_ and *ϕ*_H_, the higher overlap of HOMO and LUMO and smaller spatial separation lead to higher *J* and Δ*E*(S_1_–T_1_).^12^

6 Δ*E*(S_1_–T_1_) = *E*(S_1_) − *E*(T_1_) = 2*J*

7
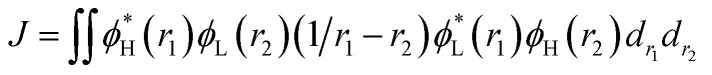


Thus, to gain a quantitative understanding, parameters such as the extent of hole–electron overlap (*S*_H/L_) and mean separation distance (Δ*R*_H–L_) of HOMO and LUMO associated with the S_1_ transitions were calculated using the Multiwfn program,^37^ and the calculated results are given in Fig. 6. As expected, a correlation between the Δ*E*(S_1_–T_1_) and *S*_H/L_ was observed for the solid state Cu(i) complexes, except for Cu(dppb)(pz_2_Bph_2_) and those with a smaller overlap *S*_H/L_ and therefore a smaller Δ*E*(S_1_–T_1_). The *S*_H/L_ overlap gradually decreases, 0.1241 > 0.1189 > 0.1119 for Cu(pop)(pz_2_BH_2_), Cu(pop)(pz_4_B), and Cu(pop)(pz_2_Bph_2_), respectively, and the corresponding Δ*E*(S_1_–T_1_) values also have a decreasing trend with 1129 > 1048 > 887 cm^−1^. For the S_1_ state of Cu(dppb)(pz_2_Bph_2_) in the solid state (*ε* = 3.66), the calculated *S*_H/L_ and Δ*R*_H–L_ values are 0.1557 and 3.06 Å, respectively, which indicate that it should have a larger Δ*E*(S_1_–T_1_); however, contrary to the theoretical analysis, it had a smaller Δ*E*(S_1_–T_1_) of 565 cm^−1^. One key factor is that this does not consider the localisation and spatial confinement of the HOMO–LUMO. This degree of spatial confinement is very crucial, which will alter the coulombic and exchange energies. Indeed, since the S_1_ state of Cu(dppb)(pz_2_Bph_2_) is more delocalised than the three other S_1_ excited states, it will exhibit the smallest Δ*E*(S_1_–T_1_). This is not addressed by the relation Δ*E*(S_1_–T_1_) ∝ *S*_H/L_.

An understanding of the degree of spatial confinement can be gained from the energetic gap (*E*_g_) between the HOMO and the LUMO and the CT excitation energy (*E*_CT_), which becomes larger with increasing localisation. According to the description, we would consequently expect that the larger *E*_g_ and *E*_CT_ would lead to the larger Δ*E*(S_1_–T_1_) as confinement increases for the Cu(i) complexes and this trend is reflected in Table 2. Here, a clear trend is found and those with a larger *E*_g_ and a larger Δ*E*(S_1_–T_1_) are observed.

### 3.3 Phosphorescence and fluorescence

For excited T_1_ states, if their phosphorescence and non-radiative decay are slow and the energy gap Δ*E*(S_1_–T_1_) is small enough, then after vibration thermalisation, a reverse of ISC back to the S_1_ can occur followed by delayed fluorescence; therefore, a detailed study of the T_1_ states is very important. We know that the degeneracy of the three spin sublevels (T_1,I_, T_1,II_, and T_1,III_) can be lifted due to the presence of zero-field splitting (ZFS) due to the SOC interaction, which can lead to quite different radiative and non-radiative properties. Yersin *et al.*^13,45^ have reported the experimental ZFS values in the range of 1 to 10 cm^−1^ for Cu(i) complex crystals. The results of the calculated ZFS parameters show that the *D* values are in the range of 0.1 to 4.7 cm^−1^ at the PCM-tuned 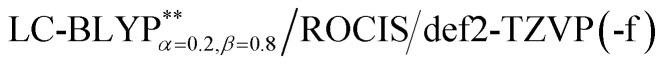 levels, consistent with the experimental values (see Table 3).

**Table 3:** Calculated spin–orbit coupling values (cm^−1^) between T_1_ and S_1_ for the four Cu(i) complexes and the ZFS tensors *D* and *E* with units in cm^−1^

Species	SOC_*x*_^a^	SOC_*y*_	SOC_*z*_	SOCC^b^	*D*	*E*
Cu(pop)(pz_2_BH_2_)	−72.74	−58.56	11.07	54.29	4.7	0.1
Cu(pop)(pz_4_B)	11.52	10.45	13.23	11.78	1.4	0.2
Cu(pop)(pz_2_Bph_2_)	−1.18	−1.16	−7.11	4.20	2.6	0.4
Cu(dppb)(pz_2_Bph_2_)	−0.50	−2.19	0.12	1.29	0.1	0.0

a
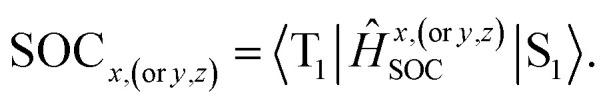

b
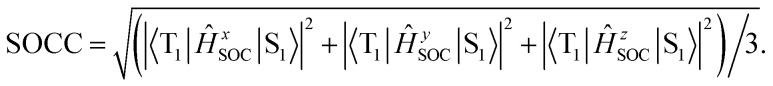

Taking into account ZFS, *k*_P_ (phosphorescence) and *k*_F_ (fluorescence) rates or (*τ*) lifetimes, have been obtained by the PCM-tuned 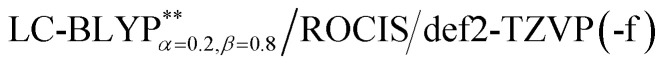 calculation at the T_1_ geometry, including SOC interactions, which are listed in Table 4. At room temperature, due to the usually small ZFS for the four Cu(i) complexes, an average emission decay time *τ*_av_ of the three sub-states can be calculated by the three individual decay times according to *τ*_av_ = 3(*τ*_I_^−1^ + *τ*_II_^−1^+ *τ*_III_^−1^)^−1^ (where *τ*_I_, *τ*_II_, and *τ*_III_ represent the emission decay times of the T_I_, T_II_, and T_III_ substates, respectively).^43^ The phosphorescence of the four Cu(i) complexes started from T_1_ and all the average radiative decay rate constants *k*_P_ are relatively slow (less than 10^3^ s^−1^), particularly for Cu(dppb)(pz_2_Bph_2_), for which the *k*_P_ was calculated to be 9.01 × 10^2^ s^−1^ because the decreased rigidity of the dppb ligand leads to a smaller *k*_P_ in comparison with the three Cu(i) molecules, including the pop ligand. The calculated means for the three phosphorescence rates are *k*_P,av_ = 7.69 × 10^3^ (*τ*_av_ = 130 μs), 1.53 × 10^3^ (*τ*_av_ = 654 μs), 2.02 × 10^2^ (*τ*_av_ = 495 μs), and 9.01 × 10^2^ s^−1^ (*τ*_av_ = 1109 μs) for Cu(pop)(pz_2_BH_2_), Cu(pop)(pz_4_B), Cu(pop)(pz_2_Bph_2_) and Cu(dppb)(pz_2_Bph_2_), respectively, and the corresponding fluorescence rates *k*_F_ (see Table 4) are in good agreement with the experimentally observed values.

**Table 4:** Computed vertical transition energies Δ*E*, oscillator strength *f*, radiative rates *k*_r_, and lifetimes *τ* of the spin sublevels at the T_1_ minimum and the corresponding experimental values in parentheses

State	Δ*E* (cm^−1^)	*f*	*k*_r_ (s^−1^)	*τ* (μs)
**Cu(pop)(pz**_**2**_ **BH**_**2**_ **)**				
T_1,I_	22343.9	1.68 × 10^−6^	5.59 × 10^2^	1789
T_1,II_	22344.0	9.80 × 10^−8^	3.26 × 10^1^	30670
T_1,III_	22348.7	6.76 × 10^−5^	2.25 × 10^4^	44
Average			*k*_P_ = 7.69 × 10^3^	*τ*_av_ (T_1_) = 130 (610)^a^
S_1_	22993.3	5.47 × 10^−2^	*k*_F_ = 2.89 × 10^7^	*τ* (S_1_) = 0.035 (0.03)^a^

**Cu(pop)(pz**_**4**_ **B)**				
T_1,I_	22728.3	1.45 × 10^−6^	5.28 × 10^2^	1894
T_1,II_	22728.5	8.28 × 10^−7^	2.85 × 10^2^	3509
T_1,III_	22729.9	1.09 × 10^−5^	3.75 × 10^3^	267
Average			*k*_P_ = 1.53 × 10^3^	*τ*_av_ (T_1_) = 654 (450)^a^
S_1_	23200.0	1.06 × 10^−2^	*k*_F_ = 3.80 × 10^6^	*τ* (S_1_) = 0.263 (0.2)^a^

**Cu(pop)(pz**_**2**_ **Bph**_**2**_ **)**				
T_1,I_	20643.3	4.07 × 10^−6^	1.16 × 10^3^	862
T_1,II_	20643.7	1.58 × 10^−5^	4.49 × 10^3^	223
T_1,III_	20646.3	1.40 × 10^−6^	3.98 × 10^2^	2513
Average			*k*_P_ = 2.02 × 10^3^	*τ*_av_ (T_1_) = 495 (480)^a^
S_1_	21380.5	3.44 × 10^−2^	*k*_F_ = 1.05 × 10^7^	*τ* (S_1_) = 0.095 (0.12)^a^

**Cu(dppb)(pz**_**2**_ **Bph**_**2**_ **)**				
T_1,I_	22576.1	2.43 × 10^−7^	8.26 × 10^1^	12106
T_1,II_	22576.1	1.73 × 10^−6^	5.88 × 10^2^	1701
T_1,III_	22576.2	5.96 × 10^−6^	2.03 × 10^3^	493
Average			*k*_P_ = 9.01 × 10^2^	*τ*_av_ (T_1_) = 1109(1200)^b^
S_1_	22948.66	2.28 × 10^−2^	*k*_F_ = 8.06 × 10^6^	*τ* (S_1_) = 0.124 (0.18)^b^

a ref. 13*a*.

b ref. 13*b*.

### 3.4 ISC and RISC

As discussed previously, owing to the small energy gap Δ*E*(S_1_–T_1_) between the S_1_ and T_1_, TADF should, in principle, be possible. However, to actually take place effectively, S_1_ has to be repopulated, which indicates that the RISC rate should be larger than the rates of radiative and nonradiative decay of the T_1_ state to the S_0_ state. In fact, the S_1_ and T_1_ states have the same electronic configurations and almost equivalent weights, and their direct SOC interaction is almost forbidden. In this case, vibronic spin–orbit coupling effects should be considered.

Intersystem crossing rate constants were calculated by considering the direct spin–orbit coupling, (*k*^dir.^_ISC/RISC_) and the vibronic spin–orbit coupling, (*k*^vib.^_ISC/RISC_) between the T_1_ and S_1_ states, as shown in Table 5. As can be seen from Tables 4 and 5, for the four Cu(i) complexes, RISC rates are approximately *k*^dir.^_RISC_ ≈ 10^6–8^ s^−1^ at 300 K, which is 4–5 orders of magnitude larger than the mean phosphorescence rate, *k*_P_ ≈ 10^2–3^ s^−1^. This indicates that the S_1_ state can be redistributed from the T_1_ state by the RISC process. At the same time, the ISC rate *k*^dir.^_ISC_ ≈ 10^9^ s^−1^ is again 2 orders of magnitude larger than the fluorescence rate *k*_F_ ≈ 10^7^ s^−1^. Based on the kinetic analysis of a three-level system proposed by Kirchhoff *et al.*,^46^ in the case of *k*^dir.^_ISC_ > *k*_F_ + *k*_IC0_ and *k*^dir.^_RISC_ > *k*_P_ + *k*_ISC0_, the S_1_ and T_1_ state populations rapidly equilibrate before decaying radiatively at room temperature, and TADF should be observable. Here, the rate constants of the internal conversion of S_1_ to S_0_ (*k*_IC0_) and of ISC from T_1_ to S_0_ were not calculated owing to the larger energy difference between S_1_ (or T_1_) and S_0_, assuming that these processes are much slower than the radiative rates and will be omitted in the following discussion.

**Table 5:** Calculated intersystem crossing rate constants (s^−1^) considering the direct spin–orbit coupling, (*k*^dir.^_ISC/RISC_) and the vibronic spin–orbit coupling, (*k*^vib.^_ISC/RISC_) between the T_1_ and S_1_ states, respectively

*T* = 300 K	*k* ^dir.^_ISC_	*k* ^dir.^_RISC_	*k* ^vib.^_ISC_	*k* ^vib.^_RISC_
Cu(pop)(pz_2_BH_2_)	4.72 × 10^9^	6.97 × 10^6^	1.39 × 10^11^	1.97 × 10^8^
Cu(pop)(pz_4_B)	5.68 × 10^9^	1.23 × 10^7^	1.74 × 10^10^	3.79 × 10^7^
Cu(pop)(pz_2_Bph_2_)	6.14 × 10^9^	2.93 × 10^7^	7.94 × 10^11^	3.78 × 10^9^
Cu(dppb)(pz_2_Bph_2_)	1.57 × 10^9^	1.04 × 10^8^	4.85 × 10^11^	1.08 × 10^10^

**Fig. 7:**
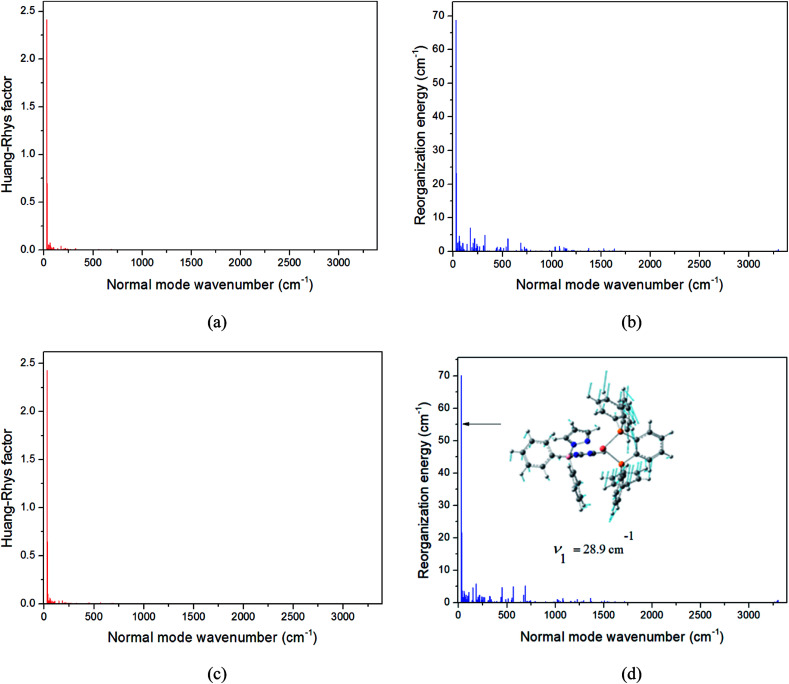
Calculated Huang-Rhys factors and reorganization energies of each normal mode on the corresponding S_1_ (a and b, red) and T_1_ (c and d, blue) potential surfaces for Cu(dppb)(pz_2_Bph_2_). The biggest displacement vector is depicted.

The first equilibrium condition, *k*^dir.^_ISC_ ≫ *k*_F_ is easily satisfied at all temperatures, while the second condition, namely *k*^dir.^_RISC_ ≫ *k*_P_, because of the strong dependence on temperature, is not. A spin-forbidden T_1_ ↔ S_1_ transition requires the effect of SOC that provides a major mechanism for RISC. SOC induces a different spin-multiplicity mixing that allows the wave function to break spin symmetry, but direct SOC between S_1_ and T_1_ states tends to zero; thus, a kind of geometrical alteration must exist, which either leads to the increase in the spin–orbit interaction or causes direct T_1_–S_1_ intersection. For the T_1_ state, among many vibration modes, only a few vibration modes play a significant role in altering the geometric structure, inducing stronger SOC interaction. In the present study, these favorite modes are called “promoting” modes, which are determined by Huang-Rhys factors and related reorganization energies of the normal modes, i (see Table S2 in ESI†). Their relationship can be expressed as follows:^47^8
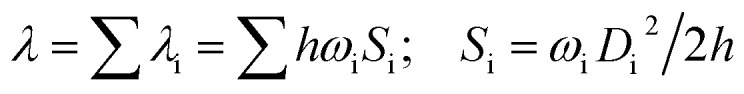
where *S*_i_ and *ω*_i_ represent the Huang-Rhys factor and the vibrational frequency for the ith normal mode, respectively; *D*_i_ is the coordinate displacement along the mode i. The extent of geometry relaxation between T_1_ and S_1_ states can be measured by the value of the Huang-Rhys factor. Here, taking the Cu(dppb)(pz_2_Bph_2_) complex as an example, the calculated *S*_i_ and *λ*_i_*versus* the normal mode i on the corresponding S_1_ and T_1_ potential surfaces are plotted in Fig. 7; those of the remaining complexes are given in Fig. S2–S4 in the ESI.† We noted that some low-frequency vibration modes have larger *S*_i_, and one of these vibration frequencies has the largest Huang-Rhys factor and reorganization energy, *i.e.*, it is that vital “promoting” mode, which corresponds to the distortion motion of the phenylene ring of the dppb ligand (see Fig. 7d). This implies that the distortion rotation of the phenylene ring can provide an important channel for conversion between T_1_ and S_1_.

**Fig. 8:**
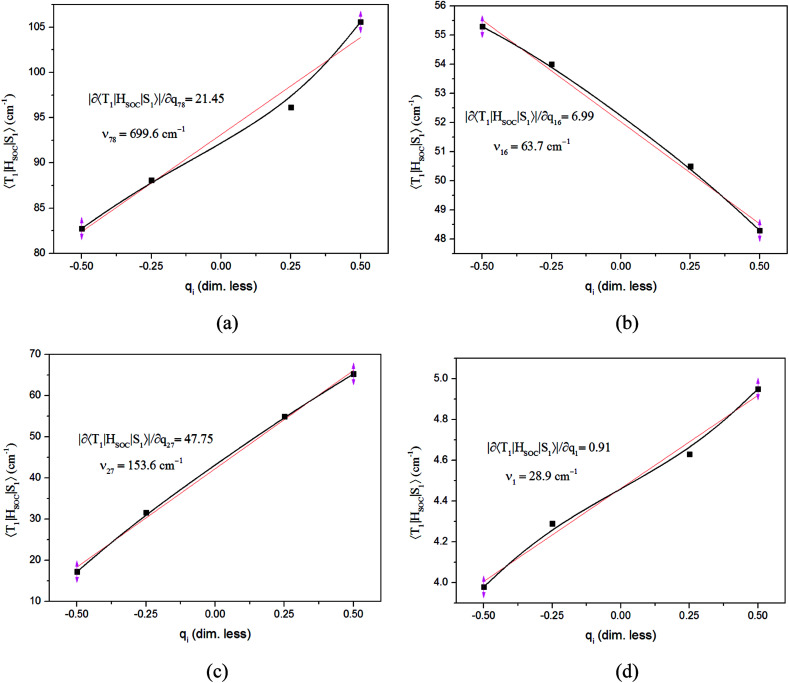
Change in the SOC (〈T_1_|*H*_SOC_|S_1_〉) upon deformation of the vital promoting vibrational normal modes in the triplet state surfaces for the RISC processes. Derivatives of SOCs with respect to the corresponding (dimensionless) coordinates at the equilibrium geometry, |∂〈T_1_|*H*_SOC_|S_1_〉|∂q_i_, and the vibrational normal frequencies, *ν*_i_, are inserted in the box. (a) Cu(pop)(pz_2_BH_2_); (b) Cu(pop)(pz_4_B); (c) Cu(pop)(pz_2_Bph_2_); (d) Cu(dppb)(pz_2_Bph_2_).

The values of component-averaged 〈T_1_|*H*_SOC_|S_1_〉 SOCs calculated along the displacement of the vital “promoting” modes with respect to the coordinates at the equilibrium geometry are illustrated in Fig. 8. For Cu(pop)(pz_2_BH_2_), Cu(pop)(pz_4_B), Cu(pop)(pz_2_Bph_2_) and Cu(dppb)(pz_2_Bph_2_), the promoting modes *ν*_78_, *ν*_16_, *ν*_27_, and *ν*_1_ with frequencies of 699.6, 63.7, 153.6 and 28.9 cm^−1^, corresponding to Huang-Rhys factors *S*_i_ = 2.07, 1.07, 4.27, and 2.42 (see Table S2 in ESI†), respectively, lead to the remarkable increase in the 〈T_1_|*H*_SOC_|S_1_〉 SOCs in the ranges of 82.5–105.2, 48.5–55.3, 16.2–65.3, and 4.0–4.9 cm^−1^, respectively. Recalling that we calculated the direct SOC to be several orders of magnitude lower, the vibronic SOC may thus be suspected to be the dominant coupling interaction here. The results show that vibronic SOC can remarkably enhance the ISC rates between T_1_ and S_1_. The largest ISC rates, *k*^vib.^_ISC_, including vibronic SOC, were actually obtained for the non-radiative transition T_1_ ↔ S_1_ and were of the order of ≈10^10–11^ s^−1^, which is about 2 orders of magnitude larger than *k*^dir.^_ISC_. Moreover, the RISC rates from T_1_ to S_1_ are affected by the same intensity. Therefore, considering the case of the vibronic SOC, TADF should be obviously observable.

## 4. Conclusions

The accuracy of the calculation level is crucial for the quantitative theoretical prediction of the photophysical properties of TADF molecules. In this study, the geometries and photophysical properties of a series of TADF Cu(i) crystal complexes have been quantitatively investigated using a methodology proposed by Sun *et al.* This method uses the combination of an optimally tuned one- and two-dimensional range-separated hybrid functional 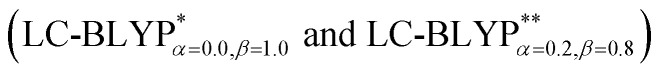 with the polarizable continuum model, in which the solid-state screening effects are achieved *via* the introduction of the solid-state dielectric constant, *ε*. When using an optimal tuning *ω* value, we find that the LC technique gives an outstanding description of the charge-transfer excited state properties. The calculated results have demonstrated that the two-dimensional tuning of the 
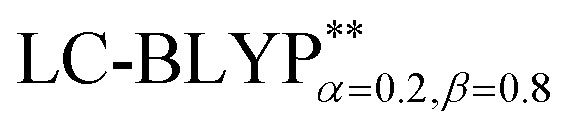
 functional provides excellent agreement with experimental data. In contrast, we find that reoptimizing the fraction of the Hartree Fock exchange in conventional hybrid functionals still yields an inconsistent description of excitation energies. For geometries, the one-dimensional tuning of the range-separated hybrid functional 
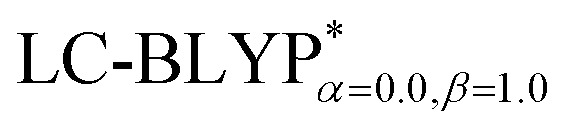
 performs slightly better than both the B3LYP and 
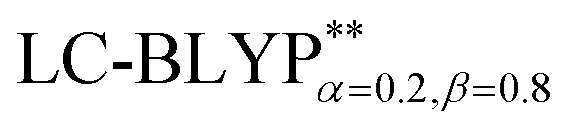
 functionals.

Photophysical radiation and non-radiation processes by combining an optimal tuning *ω* value of LC functional methods and TVCF rate theory have been comprehensively investigated for four Cu(i) complexes. At 300 K, the RISC rate was *k*^dir.^_RISC_ ≈ 10^6–8^ s^−1^, which is 4–5 orders of magnitude larger than the mean phosphorescence rate, *k*_P_ ≈ 10^2–3^ s^−1^. This indicates that the S_1_ state can be redistributed from the T_1_ state by the RISC process. At the same time, the ISC rate *k*^dir.^_ISC_ ≈ 10^9^ s^−1^ was again 2 orders of magnitude larger than the fluorescence rate *k*_F_ ≈ 10^7^ s^−1^. In the case of *k*^dir.^_ISC_ ≫ *k*_F_ and *k*^dir.^_RISC_ ≫ *k*_P_, the TADF phenomenon should be observable. Hence, the electronic-vibrational coupling activated by temperature is responsible for the occurrence of delayed fluorescence in Cu(i) complexes. It is worth noting that vibronic spin–orbit coupling can remarkably enhance the ISC rates between T_1_ and S_1_ by the vital “promoting” modes with the larger Huang-Rhys factor. These “promoting” modes correspond to the distortion motion of the phenylene ring of the pop or dppb ligands, which can provide an important pathway to decay between T_1_ and S_1_, and would be helpful for designing excellent novel TADF Cu(i) complex materials.

## Conflicts of interest

There are no conflicts to declare.

## Supplementary Material

RA-008-C8RA04978E-s001
