# Supplementary material for: Thermally activated delayed fluorescence processes for Cu(i) complexes in solid-state: a computational study using quantitative prediction
Source: RSC Adv. 2018 Aug 9;8(50):28421–32. doi: 10.1039/c8ra04978e (PMC9083942; doi:10.1039/c8ra04978e)
Supplement: RA-008-C8RA04978E-s001 [file RA-008-C8RA04978E-s001.pdf]

Electronic Supplementary Material (ESI) for RSC Advances.
This journal is © The Royal Society of Chemistry 2018

Electronic Supplementary Information (ESI)

Comprehension on Thermally Activated Delayed Fluorescence Processes for Cu(I) Complexes in Solid-State: A Computational Study Using Quantitative Prediction

Lingling Lv[*a], Kui Liu[a], Kun Yuan[a], Yuancheng Zhu[a], Yongcheng Wang[b]

[a] *College of Chemical Engineering and Technology, Tianshui Normal University, TianShui, GanSu 741001, China. E-mail: lvling100@163.com*

[b]*College of Chemistry and Chemical Engineering, Northwest Normal University, LanZhou, GanSu 730070, China. E-mail:ycwang02@163.com*

* Corresponding author: E-mail: lvling002@163.com

Table S1. Obtained vital structural parameters for Cu complexes using the different functional methods, and compared to the experimental values.

Parameters	$Cu(pop)(pz_2BH_2)$			$Cu(pop)(pz_4B)$			$Cu(pop)(pz_2Bph_2)$			$Cu(dppb)(pz_2Bph_2)$		
	B3LYP	LC-BLYP*	Exp. value	B3LYP	LC-BLYP*	Exp. value	B3LYP	LC-BLYP*	Exp. value	B3LYP	LC-BLYP*	Exp. value
R(Cu1–P1)	2.3606	2.2919	2.2704	2.3957	2.3127	2.2925	2.4196	2.3322	2.2957	2.3457	2.2858	2.2571
R(Cu1–P2)	2.3508	2.2838	2.2348	2.3348	2.2766	2.2319	2.3245	2.2702	2.2203	2.3148	2.2778	2.2562
R(Cu1–N1)	2.1002	2.0512	2.0501	2.0867	2.0455	2.0444	2.0812	2.0407	2.0290	2.0555	2.0196	2.0182
R(Cu1–N3)	2.1096	2.0535	2.0355	2.0917	2.0404	2.0251	2.0799	2.0362	2.0197	2.0519	2.0168	2.0112
∠P1Cu1P2	112.57	112.62	112.15	112.77	112.97	110.26	111.71	112.52	109.20	88.85	90.26	
∠N1Cu1N3	94.01	95.59	96.14	92.95	95.19	95.51	93.92	96.26	96.45	94.15	95.66	
MAD[a]	0.0831	0.0224		0.0787	0.0203		0.0851	0.0286		0.0563	0.0143	
MAD[b]	1.41	0.51		2.535	1.195		2.52	1.780				

MAD[a]: Mean absolute deviations of bond lengths; MAD[b]: Mean absolute deviations of bond angles. The MAD values are calculated with respect to the corresponding experimental values,

$$MAD = 1/n \sum_1^n \left| R_{cal.} - R_{exp.} \right| \cdot$$

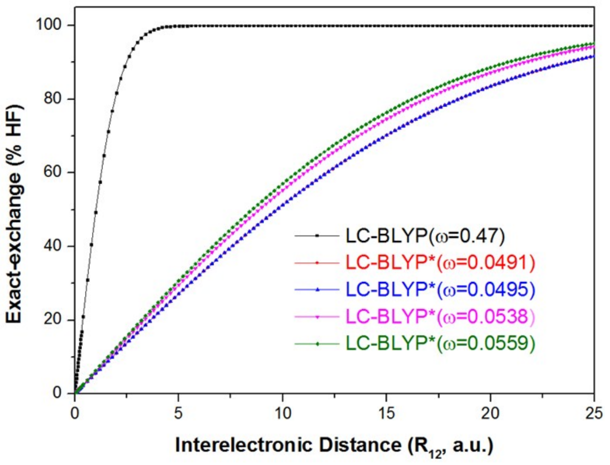

Figure S1. Percentage of exact-exchange (%HF) included as a function of the interelectronic distance (R_{12}) for LC-BLYP functional in the solid state.

Table S2. The vital promote vibrational normal modes, and the corresponding Huang-Rhys factor (S_i), reorganization energy (λ_i) in the triplet state surfaces for the RISC processes.

Cu(pop)(pz_2BH_2)	Cu(pop)(pz_4B)	Cu(pop)(pz_2Bph_2)	Cu(dppb)(pz_2Bph_2)
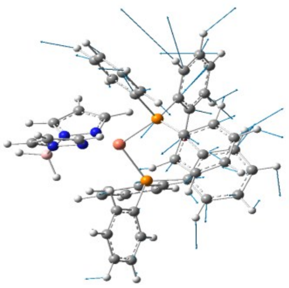	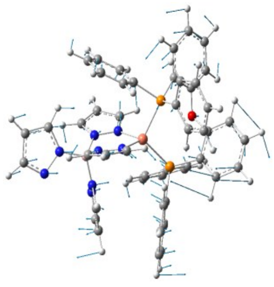	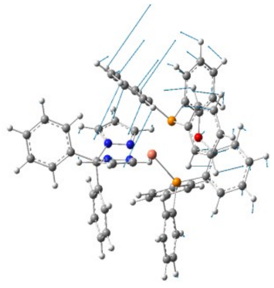	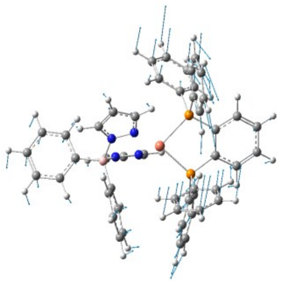
ν_{78} = 699.6 cm^{-1}<br>S_{78} = 2.07<br>λ_{78} = 1447.86 cm^{-1}	ν_{16} = 63.7 cm^{-1}<br>S_{16} = 1.07<br>λ_{16} = 68.63 cm^{-1}	ν_{27} = 153.6 cm^{-1}<br>S_{27} = 4.27<br>λ_{27} = 656.14 cm^{-1}	ν_1= 28.9 cm^{-1}<br>S_1 = 2.42<br>λ_1 = 70.04 cm^{-1}

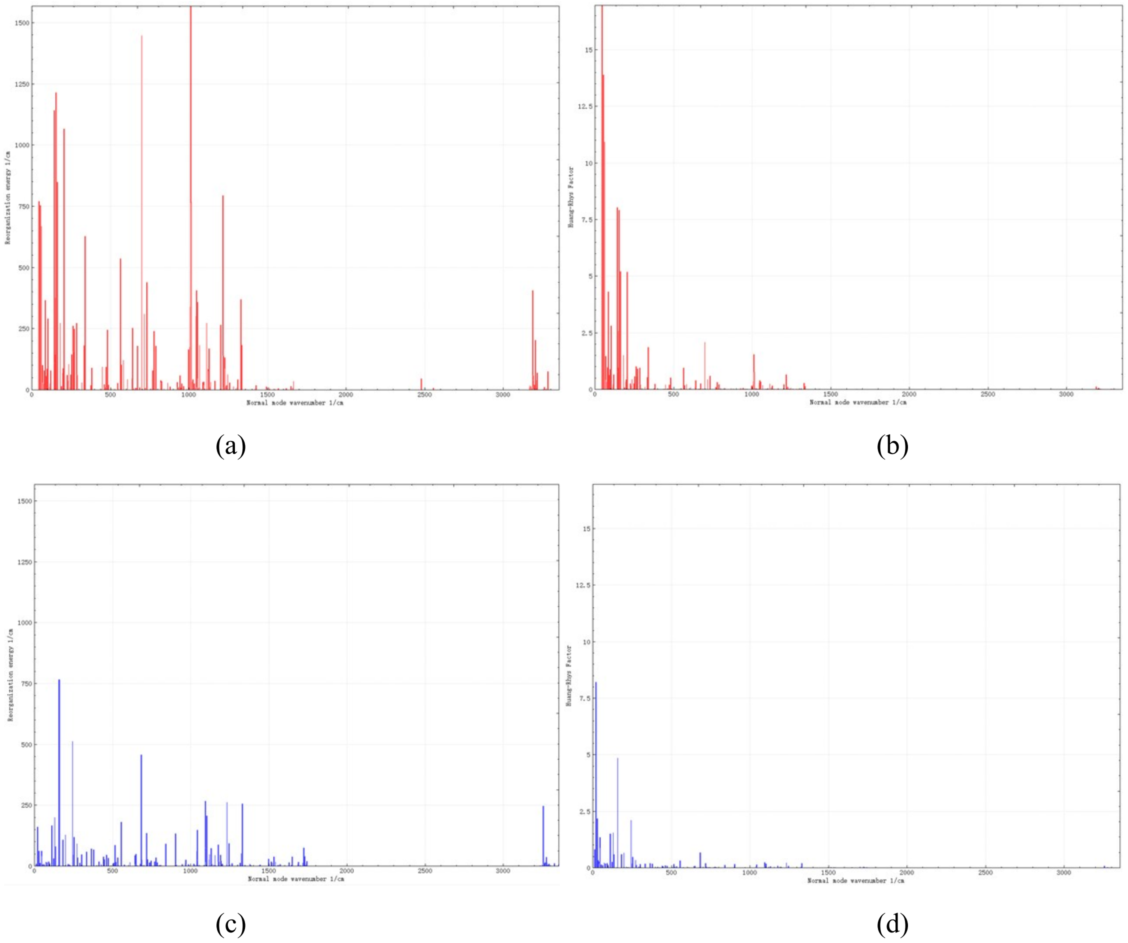

Figure S2. Calculated reorganization energies and Huang-Rhys factors versus the normal modes in term of the corresponding T_1 (a, b, red) and S_1 (c, d, blue) potential surfaces for Cu(pop)(pz_2BH_2) at the PCM-tuned LC-BLYP* level .

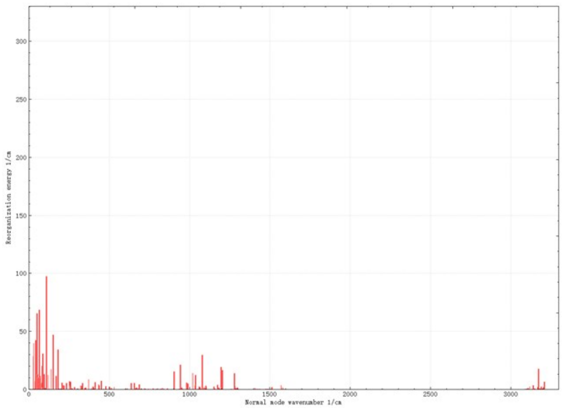

(a)

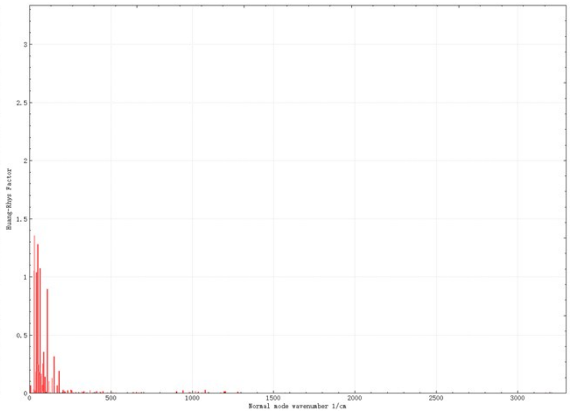

(b)

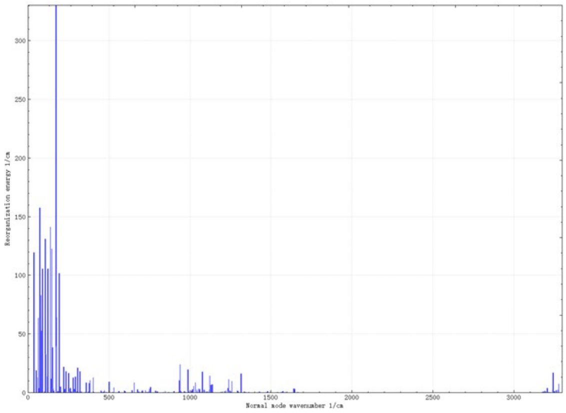

(c)

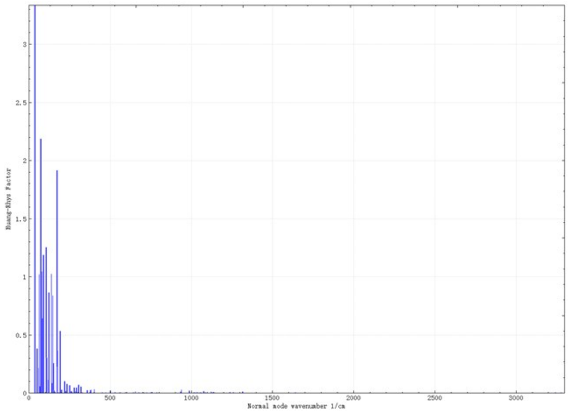

(d)

Figure S3. Calculated reorganization energies and Huang-Rhys factors versus the normal modes in term of the corresponding T_1 (a, b, red) and S_1 (c, d, blue) potential surfaces for Cu(pop)(pz_4B) at the PCM-tuned LC-BLYP* level .

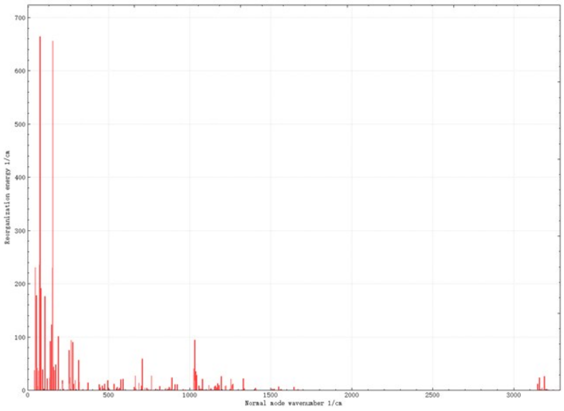

(a)

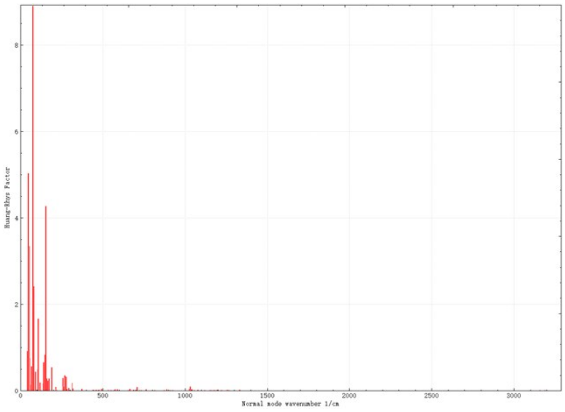

(b)

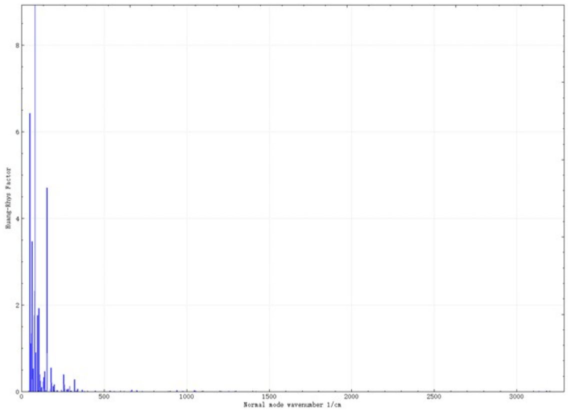

(c)

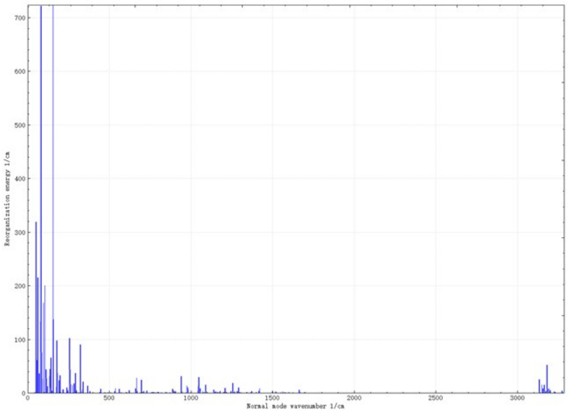

(d)

Figure S4. Calculated reorganization energies and Huang-Rhys factors versus the normal modes in term of the corresponding T_1 (a, b, red) and S_1 (c, d, blue) potential surfaces for Cu(pop)(pz_2Bph_2) at the PCM-tuned LC-BLYP* level .

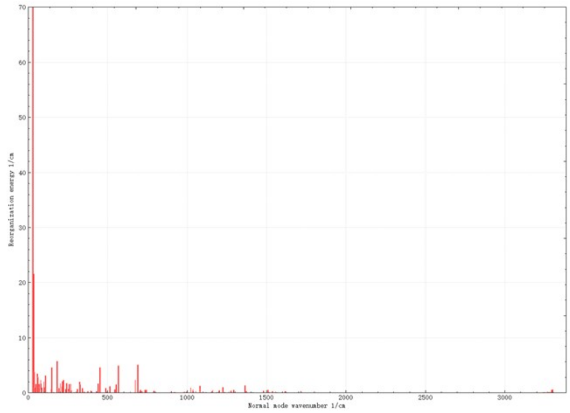

(a)

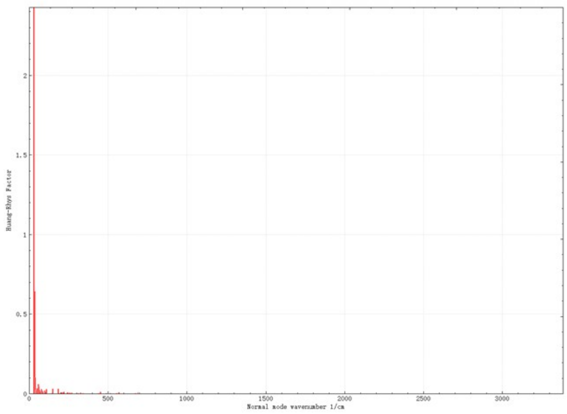

(b)

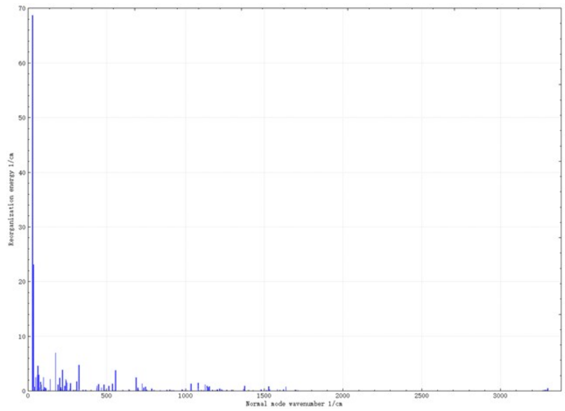

(c)

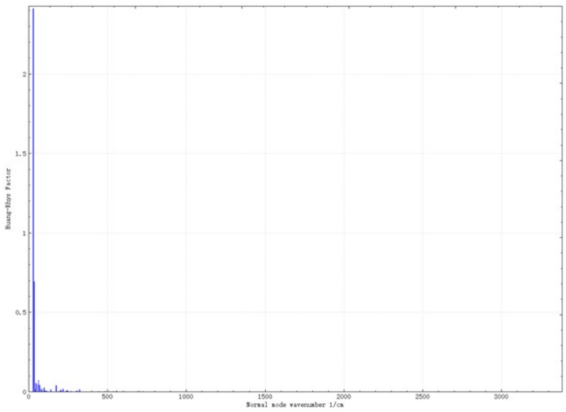

(d)

Figure S5. Calculated reorganization energies and Huang-Rhys factors versus the normal modes in term of the corresponding T_1 (a, b, red) and S_1 (c, d, blue) potential surfaces for Cu(dppb)(pz_2Bph_2) at the PCM-tuned LC-BLYP* level .
